# Steady-state first-pass perfusion (SSFPP): A 3D TWIST in myocardial first-pass perfusion imaging

**DOI:** 10.1186/1532-429X-14-S1-P251

**Published:** 2012-02-01

**Authors:** Shivraman Giri, Hui Xue, Abdul Wattar, Yu Ding, Randall M Kroeker, Gerhard Laub, Peter Kellman, Sven Zuehlsdorff, Subha V Raman, Orlando P Simonetti

**Affiliations:** 1 The Ohio State University, Columbus, OH, USA; 2 Siemens Corporate Research, Princeton, NJ, USA; 3 Siemens Healthcare, Winnipeg, MB, Canada; 4 Siemens Healthcare, San Francisco, CA, USA; 5 Siemens Healthcare, Chicago, IL, USA; 6 National Institutes of Health, Bethesda, MD, USA

## Summary

A new approach to myocardial first-pass perfusion imaging is presented; this technique, called SSFPP, is based on 3D SSFP sequence whereby magnetization is maintained in constant steady-state, while data acquisition is gated to diastole. This results in high SNR and CNR, and other characteristics that can potentially mitigate dark rim artifacts. The high-blood myocardial contrast of SSFP allows automatic segmentation, which, combined with registration, facilitates image analysis.

## Background

Although introduced in 1990, myocardial first-pass perfusion imaging has not yet become a routine diagnostic tool, primarily because of insufficient image quality, insufficient coverage, and dark rim artifacts (DRA). Current techniques rely on a saturation recovery (SR) preparation for T1 contrast, resulting in poor SNR, low efficiency, and k-space modulation during SR. Further, the post-processing of these images is tedious. In this work, we propose an alternative perfusion imaging technique, called Steady-State First Pass Perfusion (SSFPP).

## Objective

To develop a new 3D first-pass perfusion imaging technique that can potentially address the limitations of current methods.

## Theory

SSFPP is a 3D SSFP sequence in which the magnetization is maintained in constant steady-state while the data acquisition is gated to diastole. The SNR and CNR are similar to those in SSFP cine imaging, allowing the use of automatic segmentation algorithms. Furthermore, the tissue contrast is dependent on T1/T2; serendipitously, this causes blood signal to remain almost constant, whereas the myocardial signal exhibits a nearly linear correlation with contrast agent concentration. Maintenance of steady-state throughout data acquisition avoids k-space modulation, and the elimination of saturation recovery time increases data acquisition efficiency by reducing deadtime.

## Methods

SSFPP was implemented on a 1.5T scanner (Avanto, Siemens). RF pulse (time-bandwidth product = 10, flip angle ~40°.) was optimized for 3D slab excitation profile. Other parameters: resolution ~2.2x2.8x8mm^3^, matrix 160x103x6, slab oversampling 33.3%, TR = ~2.7 ms, Multihance (0.1 mmol/kg). 3D K-space was acquired using parallel imaging (GRAPPA, rate=3, 24 intrinsic reference lines, 32 channel phased array coil (QED LLC)) and TWIST acquisition scheme; for the latter, a central 4% of k-space was updated every frame, whereas the peripheral region was undersampled at 33%, leaving a “temporal footprint” of 3 heart beats. Acquisition time per 3D frame was ~300-340 ms. Images were acquired in three healthy subjects during contrast agent injection to evaluate feasibility of this new method.

Non-rigid registration, optimized for dynamically varying contrast, was used for three-dimensional motion-correction prior to automated contouring of endo and epicardial borders.

Pixel-wise contrast enhancement ratio (CER) maps were computed for each frame, where each pixel is given by: (S_n_ - S_baseline_)/S_baseline_; time intensity curves (TIC) of these CER images were used for semi-quantitative analysis.

## Results

**Figure 1:**
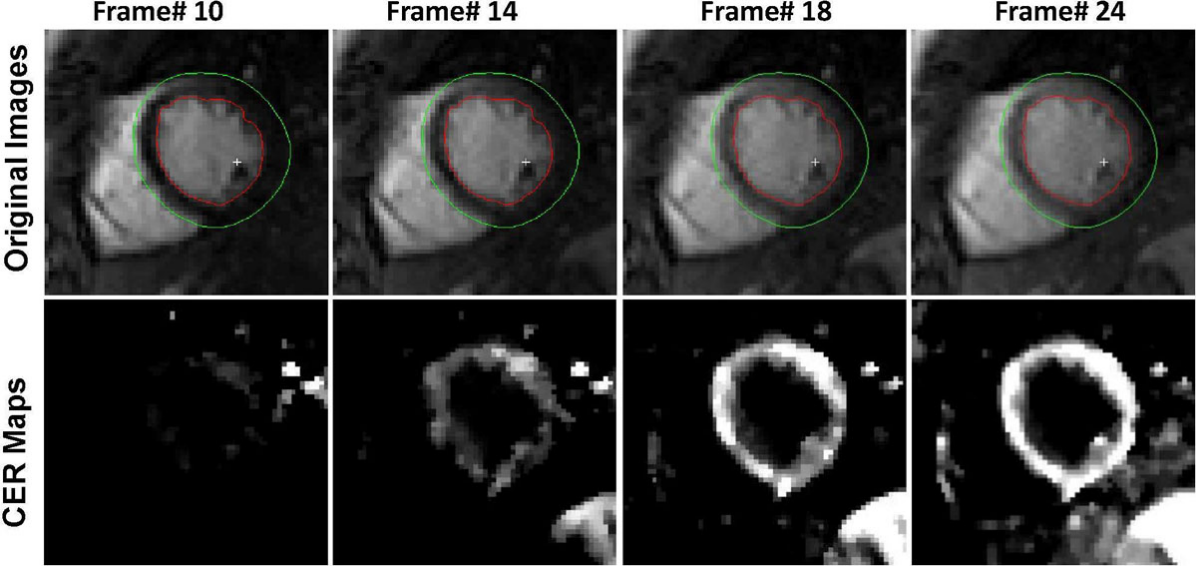
Select myocardial frames during upslope (top row) and their corresponding pixel-wise contrast enhancement ratio (CER) maps. Note that the SNR and CNR of original frames permits automatic contouring that, along with registration, can facilitate image analysis. In this case, the contours were drawn automatically in the first frame, and were then propagated to other frames. Also note that bloodpool signal remains almost constant with changing [Gd] and is therefore suppressed in CER map.

**Figure 2:**
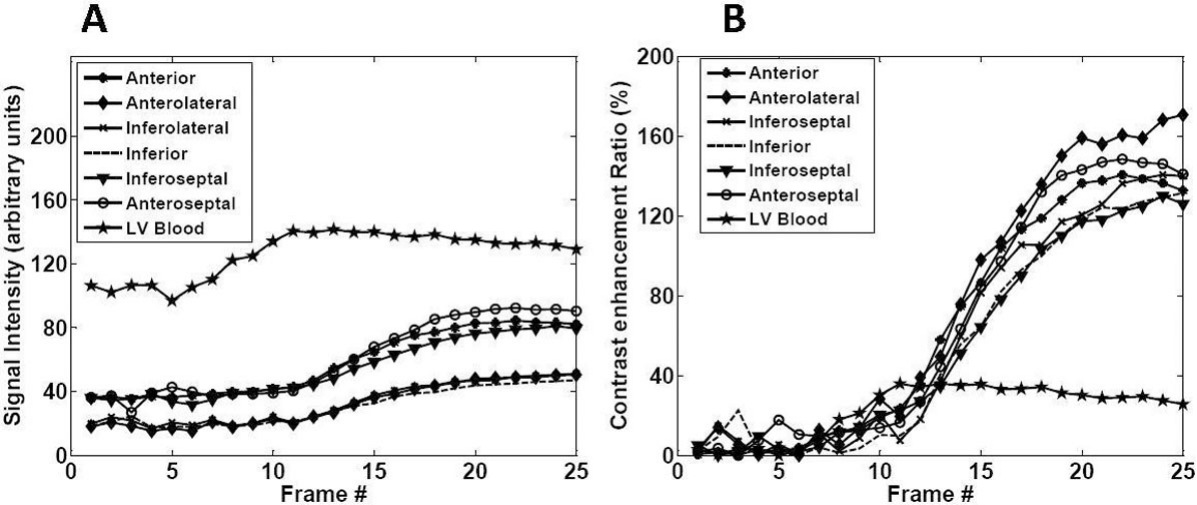
Signal intensity (A) and contrast enhancement ratio (CER) (B) curves for left ventricular blood pool and six myocardial segments for the slice shown in Figure 1. Note that the blood signal stays nearly constant compared with myocardium. Also note that the signal intensity is affected by the use of phase array coils, with the segments closer to the coil having higher signal; this is partly corrected for in the CER maps.

Figures 1 and 2 show images and TICs from a SAX slice of one subject. Similar results were noted in other two subjects.

## Conclusions

3D SSFPP avoids many of the suspected causes of DRA and could potentially mitigate this problem. Steady-state imaging provides high SNR and CNR that, combined with image registration, can facilitate effective perfusion quantification.

## Funding

The project is partially supported by Award Number R01HL102450 from the National Heart, Lung, and Blood Institute.

